# Epidemic of lumpy skin disease in Pakistan

**DOI:** 10.1002/vms3.1037

**Published:** 2023-01-16

**Authors:** Govinda Khatri, Aneesh Rai, Aashish, Shahzaib, Saqlain Hyder, Priya, Mohammad Mehedi Hasan

**Affiliations:** ^1^ Dow University of Health Sciences Karachi Pakistan; ^2^ Department of Biochemistry and Molecular Biology Faculty of Life Science Mawlana Bhashani Science and Technology University Tangail Bangladesh

## Abstract

Lumpy skin disease (LSD) is a viral disease that affects farm animals including water buffalo. It is caused by the contagious LSD virus, a member of the Poxiviridae family's Capripox genus. Skin sores are thought to be the most common site of infection since the virus may live for lengthy periods in lesions or scabs. The first clinical indications of LSD were described in Zambia, in 1929. Pakistan has also been afflicted by LSD, with a high number of animals infected at many cattle ranches in Karachi, 190,000 cases of LSD have been reported nationwide, with greater than 7500 deaths attributable to the illness. LSD has a huge influence on Pakistan's economic status, resulting in the loss of cattle and a decrease in milk output. The Ministry of Research and National Food Safety in Pakistan has formed a working group to create a framework for controlling the spread of LSD in cattle and buffalo. Official and private veterinarians, both field and slaughterhouse, veterinary students, farmers, cattle merchants, cattle truck drivers, and artificial inseminators should all participate in awareness efforts.

## Graphical Abstract

This article aims to detail the virus that causes lumpy skin disease (LSD), its diagnosis, symptoms, treatment and the economic and health impact of LSD in Pakistan. It also discusses the preventative measures taken by the government of Pakistan and the proposals for controlling additional increases in cases.

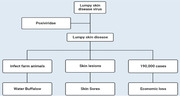

## 1 INTRODUCTION

The lumpy skin disease (LSD) is a viral infection of farm animals and water buffalo. It is prevalent in most African and Middle Eastern nations. It is caused by the lumpy skin disease virus (LSDV), which belongs to the genus Capri pox of the poxiviridae family. The virus is transmitted mostly by arthropod vectors, i.e., insects, but it is also believed to be transmitted via direct contact with the infected animal, contaminated feed and water. Skin lesions are considered the main source of infection since the virus persists in lesions or crusts for a long time (Namazi & Khodakaram Tafti, 2021).

The diagnosis of LSD is made by histopathological evidence, virus isolation or PCR. The disease may be confused with cows’ pseudo‐LSD caused by a herpes virus. The two diseases can be clinically differentiated by PCR (Amin et al., 2021). Although there are no specific antiviral drugs for LSDV, the only treatment option is livestock supportive care. Secondary infections of the skin can be treated with NSAIDs or antibiotics. The only method of control is preventive, i.e., vaccination (Babiuk, 2018).

Due to the recent outbreaks in the neighboring countries like Malaysia and Thailand (Azeem et al., 2022), and the movement of livestock from and to Pakistan, the LSD can cause an outbreak in Pakistan. Historically being free of the LSDV, the cases are now reported in Pakistan with more than 20,000 animals affected in only Sindh. Karachi is the city with the most reported cases, about 54 animals had died in the province, while 4751 had recovered across the province (DAWN, 2022b). Since the disease's breakout, 190,000 cases of LSD have been reported nationwide, with greater than 7500 deaths attributable to the illness. However, the recovery rate emerged to be satisfactory, with over 141,000 animals recovered. Furthermore, Pakistan is already dealing with COVID‐19 disasters; the present COVID‐19 pandemic has had serious ramifications for the country's healthcare sector (Awan et al., 2022; Khatri et al., 2022), and further crises like LSD might have devastating consequences for Pakistan's economy and the health care system. LSD has spread throughout the country, with cases reported in Sindh (Daily Times, n.d.), Punjab, Balochistan (DAWN, 2022a) and Khyber Pakhtunkhwa (DAWN, 2022a). So far, 74,590 animals have been infected in Khyber Pakhtunkhwa, 53,668 in Sindh, 35,000 in Punjab, 22,225 in Balochistan, and 6351 in Azad Jammu and Kashmir.

This article aims to detail the virus that causes LSD, its diagnosis, symptoms, treatment and the economic and health impact of LSD in Pakistan. It also discusses the preventative measures taken by the government of Pakistan and the proposals for controlling additional increases in cases.

## 2 DISCUSSION

In 1929, the first clinical symptoms of LSD were reported in Zambia. Initially, LSD symptoms were thought to be the result of poisoning or allergic reactions to insect bites. Between 1943 and 1945, the same clinical symptoms appeared in the Republic of South Africa, Zimbabwe and Botswana. In these epidemics, the infectious origin of the disease was discovered (Farah Gumbe, 2018). Until 1988, the infection was restricted to greater Africa, with subsequent spread to the Middle East, Eastern Europe, and the Russian Federation. New cases were reported in South and East Asia in 2019, as the outbreak spread further (Das et al., 2021).

The disease invaded China across the Kazakh border in 2019, with 65 affected animals reported. It found its way into India shortly after 66 cases were recorded for the first time in Bangladesh in July 2019 (Khan et al., 2021). Currently, the extremely contagious disease LSD is affecting a large portion of Southeast Asian animals at a rapid rate. Bangladesh was the first country on the Asian continent to report a case of LSD, followed by China, India, Nepal, Bhutan, Vietnam, Hong Kong and Myanmar (Das et al., 2021). Pakistan has also been affected by LSD as a large number of animals have been infected at several cattle farms in Karachi and more than 200 animals have died so far due to it (Nazir, 2022).

LSD has serious economic implications, and is associated with reduced milk production, temporary or permanent infertility in cows and bulls, weight loss, loss of traction, abortion, skin damage and death. In addition, vaccinating, campaigning and disposing of affected animals are costly and can further impact the economy. The economic impact of LSD can be determined by calculating direct production losses, such as milk loss, mortality loss, tensile strength loss and indirect impacts, such as cost control (Molla et al., 2017).

LSD's rise in a country like Pakistan, which is primarily reliant on agriculture and has the world's second‐largest cow population, would be disastrous. Pakistan's livestock population includes 49.6 million cattle and 41.2 million buffaloes, with annual increases of 3.1 million and 1.2 million cattle and buffaloes, respectively. Livestock, the country's largest subsector of agricultural output, accounts for Rs.1466 billion of Pakistan's GDP. Approximately 8 million households are actively involved in the cattle trade, which provides 35%–40% of their income. The economic consequences of the rise of this devastating disease on a nation with an already fragile and diminishing economy can be severe and long‐lasting, due to the imposition of anticipated blockades on livestock trade and a severe decline in the rural economy of eight million families (Khan et al., 2021). As LSDV has impacted more than 190,000 animals across the nation, this outbreak would have a significant impact on Pakistan's economic situation. Pakistan is the 3rd largest milk‐producing country in the world, with over 47 million tons of milk produced per year (Abdus Sattar, 2021). LSD‐infected cows will not be able to produce milk for several months and even after recovering it will take time to regain the same production level, which overall will impact the country's economy (Molla et al., 2017). The financial losses that Pakistan will face due to LSD can be estimated by comparing the stats of Ethiopia which included USD 375 overall median financial loss per dead animal, and USD 141 financial loss of Milk per affected cow (Molla et al., 2017).

During the global COVID‐19 pandemic, Pakistan has been severely impacted; there have been over 1.5 million confirmed COVID‐19 cases in Pakistan, with 30,340 deaths (WHO, 2022). The economic growth of dairy farms has been severely impacted by COVID‐19 around the world due to a drop in milk production and restrictions on milk sales. There has also been a shortage of veterinary medicines at the veterinary pharmacy due to hoarding, and most diagnostic laboratories are focused on COVID‐19, resulting in a limited diagnosis of animal diseases (Hussain et al., 2020).

Emerging an epidemic in midst of a pandemic will cause severe problems such as already low production of milk and dairy products will be further lowered and unable to meet the demands. Additionally, already a shortage of veterinary drugs will lead to the inability to treat the secondary diseases in animals due to the LSDV. Pakistan will have to spend a lot of money to eradicate the disease as in the past Israel had to spend USD 750,000 to control the initial outbreak of LSD. Although the vaccination will cost a lot of expenses economic benefits gained by controlling LSD with vaccination can be taken into account by comparing the reduction in economic loss with the expenditure for the vaccine (Molla et al., 2017).

## 3 CALL OF ACTION AND RECOMMENDATIONS

A task force has been formed by Pakistan's Ministry of National Food Security and Research to design a framework for preventing the development of LSD in cattle and buffaloes. As per Dr. Mohammad Akram, Animal Husbandry Commissioner, to combat the spread of LSD 500,000 vaccinations had to be imported. Though vaccines must be imported, as a temporary substitute “Goat Pox” vaccine is being used, which has been demonstrated to be effective (DAWN, 2022c).

In addition, a ban on the livestock market has been imposed to prevent the spread of the disease. Cattle owners should separate sick animals from healthy ones and use anti‐mosquito spray frequently to protect their animals from LSD, as directed by the Live Stock Department. Special teams will be sent to Diary farms to vaccinate cattle against the viral disease. Various changes have been implemented in the country for the prevention of the disease. The farmers of Pakistan do keep the infected cattle separate from the healthy ones and furthermore, they go to the veterinarian to get a prescription of drugs for cattle. Farmers also have been eliminated sources of vector that as stagnant water by increasing drainage. The authorities of Pakistan are raising awareness of the disease in various regions. In Sindh, the local Government dispatched 30,000 vaccines which were administered to cattle in Karachi on March 2022 (Gavi, Vaccine Alliance, n.d.).

Cattle movement within the country and throughout borders should be carefully restricted, if not altogether outlawed. Cattle movements that are authorized should be accompanied by a veterinarian certificate that includes all information about the animals’ origins as well as animal health guarantees. To reduce the danger of disease transmission by vectors, cattle should be treated with insect repellents regularly. Reducing the number of vectors in and around livestock by limiting vector breeding grounds such as sources of stagnant water, manure and slurry, and increasing drainage on farms are sustainable, cost‐effective and environmentally friendly methods. Awareness campaigns should be carried out for official and private veterinarians, both field and slaughterhouse, veterinary students, ranchers, ranchers, truck drivers, and artificial inseminators. Active and passive clinical monitoring, as well as laboratory testing of blood samples, nasal swabs and skin biopsies acquired from suspected cases, are used in surveillance programs. On affected farms, trucks, premises and possibly contaminated locations, thorough cleaning and disinfection with appropriate materials should be conducted. Personnel should be disinfected as well (Fao, 2017). Vaccination is the most effective method to reduce the outbreaks of LSD. Only a live attenuated vaccine is commercially available. I is estimated to be 75% effective. Vaccination should be done in all areas uniformly to avoid areas with large numbers of unvaccinated farms.

## 4 CONCLUSION

LSD is a lethal disease in cattle, yet it does not affect the human body. A recent epidemic of LSD in Pakistan during the COVID‐19 damaged Pakistan's economic condition owing to animal deaths. Pakistan's Ministry of National Food Security and Research has organized a working group to develop a framework to control the development of LSD in cattle and buffalo. The government should adopt a variety of measures to restrict the spread of LSD.

## AUTHOR CONTRIBUTIONS

Govinda Khatri: *Conceptualization; writing–original draft; writing—review & editing*. Aneesh Rai and Aashish: *Writing—original draft; writing—review & editing*. Shahzaib, Saqlain Hyder and Priya: *Writing—original draft*. Mohammad Mehedi Hasan: *Conceptualization; supervision; writing—review & editing*.

## CONFLICT OF INTERESTS

The author declares that there is no conflict of interest that could be perceived as prejudicing the impartiality of the research reported.

## FUNDING INFORMATION

The authors received no specific funding for this work.

### PEER REVIEW

The peer review history for this article is available at https://publons.com/publon/10.1002/vms3.1037

## Data Availability

Data sharing is not applicable to this article as no new data were created or analyzed in this study.
